# A case of macrophage activation syndrome in a child with systemic juvenile idiopathic arthritis

**DOI:** 10.1186/1546-0096-12-S1-P230

**Published:** 2014-09-17

**Authors:** Aida Akhenbekova, Nataly Urasalieva, Elena Kustova

**Affiliations:** 1 Rheumatology, Scientific center of pediatrics and children surgery, Almaty, Kazakhstan

## Introduction

Systemic type of juvenile idiopathic arthritis (JIA) is highly active autoimmune process. One of its severe complications is macrophage activation syndrome, seriously influenced the outcome of the disease. The aim of the report is to reveal the macrophage activation syndrome during the onset of JIA, before treatment.

## Objectives

A patient 1.3 years old, in the onset of JIA.

## Methods

All routine analyses, including the blood test, biochemical serum determination of antinuclear antibodies to autoimmune hepatitis, markers of viral hepatitis, anitbodies to Epstein-Barr virus, CMV, herpes infection, toxoplasmosis, chlamydia, mycoplasmosis, HIV were performed. Instrumental methods included: chest radiography, tomography t, echocardiography, electrocardiography.

## Results

History of the disease: the beginning of the disease was characterized with erythematosis rash and febrile temperature. Infectious diseases were excluded. Therapy with 10 mg of prednisolone per day had a temporary positive effect. However, varicella relapse with recurrent fever took place 3 weeks after.

The following symptoms have been reported: fever up to 39.0^0^C, a rash on the upper limbs, thighs, legs, swelling of the knee joints, morning stiffness, vasculitis in the form of erythematous spots all over the body, carditis, pneumonitis, enterocolitis , lymphadenopathy . In blood – leukocytosis - 28.2 thousand with a shift to young leukocyte cells, eritrocyte sedimentation - 48 mm/h ; CRP - 78.2mg, rheumatoid factor, antinuclear antibodies - negative, normal range of ferritine. High dose methylprednisolone therapy – not effective. Deterioration due to bronchopulmonary infection. Chest radiography and tomography revealed bronchiolitis, bronhoobstructive syndrome and respiratory insufficiency. Immunosuppressive therapy combined with antibiotic therapy, after which autoimmune hepatitis (ALT - 4898 IU/l, AST -3100 IU/l, ferritin – 3600 mkg/l , LDH -1200 IU /l, Bil total - 116 mmol/l .However,clinicall situation became much better: normal body temperature, relief of rashes and respiratory failure. Leicocytosis decreased to 17x10^3^. Macrophage activation syndrome was possible, but there was no decrease of platelets. Therapy included 2 mg/kg of prednisolone orally, detoxication, hepatoprotectors therapy, correction of hemostasis. Within 3 weeks there was a slow decline in level of transaminases, ferritin, total bilirubin what gave opportunity to reduce doze to 1,3 mg/day.

## Conclusion

Analyze of the disease symptoms showed that the autoimmune hepatitis had been developed because of the macrophage activation syndrome after using the antibacterial drug. Probably, such pathology reaction of the immune system is genetically determined. It was unexpectedly, that oral usage of prednisolone would show better efficiency in compare with pulse therapy. Our case illustrates the difficulty of MAS diagnostics in children with JIA and can be the result of immune system hyperactivity caused by unknown reasons. Mechanisms of MAS syndrome require to be studied in detail and included in early diagnostic criteria.

## Disclosure of interest

None declared.

